# Necrosis binding of Ac-Lys^0^(IRDye800CW)-Tyr^3^-octreotate: a consequence from cyanine-labeling of small molecules

**DOI:** 10.1186/s13550-021-00789-4

**Published:** 2021-05-10

**Authors:** Marcus C. M. Stroet, Bianca M. Dijkstra, Sebastiaan E. Dulfer, Schelto Kruijff, Wilfred F. A. den Dunnen, Frank A. E. Kruyt, Rob J. M. Groen, Yann Seimbille, Kranthi M. Panth, Laura Mezzanotte, Clemens W. G. M. Lowik, Marion de Jong

**Affiliations:** 1 Department of Radiology and Nuclear Medicine/Molecular Genetics, Erasmus Medical Centre, ’s-Gravendijkwal 230, 3015 CE Rotterdam, The Netherlands; 2 Department of Molecular Genetics, Erasmus MC, Rotterdam, The Netherlands; 3 Department of Neurosurgery, University of Groningen, University Medical Center Groningen, Groningen, The Netherlands; 4 Department of Surgery, University of Groningen, University Medical Center Groningen, Groningen, The Netherlands; 5 Department of Pathology and Medical Biology, University of Groningen, University Medical Center Groningen, Groningen, The Netherlands; 6 Department of Medical Oncology, University of Groningen, University Medical Center Groningen, Groningen, The Netherlands; 7 CHUV Department of Oncology, University of Lausanne, Lausanne, Switzerland

**Keywords:** 800CW-TATE, Molecular fluorescence-guided surgery, Somatostatin receptor subtype 2, Fluorescent molecular probes, Necrosis-avidity

## Abstract

**Background:**

There is a growing body of nuclear contrast agents that are repurposed for fluorescence-guided surgery. New contrast agents are obtained by substituting the radioactive tag with, or adding a fluorescent cyanine to the molecular structure of antibodies or peptides. This enables intra-operative fluorescent detection of cancerous tissue, leading to more complete tumor resection. However, these fluorescent cyanines can have a remarkable influence on pharmacokinetics and tumor uptake, especially when labeled to smaller targeting vectors such as peptides. Here we demonstrate the effect of cyanine-mediated dead cell-binding of Ac-Lys^0^(IRDye800CW)-Tyr^3^-octreotate (800CW-TATE) and how this can be used as an advantage for fluorescence-guided surgery.

**Results:**

Binding of 800CW-TATE could be blocked with DOTA^0^-Tyr^3^-octreotate (DOTA-TATE) on cultured SSTR_2_-positive U2OS cells and was absent in SSTR_2_ negative U2OS cells. However, strong binding was observed to dead cells, which could not be blocked with DOTA-TATE and was also present in dead SSTR_2_ negative cells. No SSTR_2_-mediated binding was observed in frozen tumor sections, possibly due to disruption of the cells in the process of sectioning the tissue before exposure to the contrast agent. DOTA-TATE blocking resulted in an incomplete reduction of 61.5 ± 5.8% fluorescence uptake by NCI-H69-tumors in mice. Near-infrared imaging and dead cell staining on paraffin sections from resected tumors revealed that fluorescence uptake persisted in necrotic regions upon blocking with DOTA-TATE.

**Conclusion:**

This study shows that labeling peptides with cyanines can result in dead cell binding. This does not hamper the ultimate purpose of fluorescence-guided surgery, as necrotic tissue appears in most solid tumors. Hence, the necrosis binding can increase the overall tumor uptake. Moreover, necrotic tissue should be removed as much as possible: it cannot be salvaged, causes inflammation, and is tumorigenic. However, when performing binding experiments to cells with disrupted membrane integrity, which is routinely done with nuclear probes, this dead cell-binding can resemble non-specific binding. This study will benefit the development of fluorescent contrast agents.

**Supplementary information:**

The online version contains supplementary material available at 10.1186/s13550-021-00789-4.

## Background

There is a growing interest in the development of optical molecular contrast agents for fluorescence-guided surgery [1, 2]. In most cases, successful targeting vectors from nuclear imaging agents are repurposed, either by replacing the radiolabeling part with a fluorescent tag for single modal imaging, or by adding both a radioactive and a fluorescent chemical moiety for bi-modal imaging. These approaches have already yielded ample probes, with some explored in clinical trials [3, 4]. Most of these tracers are based on antibodies, which are relatively big molecules on which the molecular burden of both tags has little effect on the affinity for the targeted epitope [1, 5]. Currently, a growing body of peptides and small molecules is advanced as targeting vectors for optical imaging. However, fluorescent tags can have a drastic influence on the pharmacokinetic properties of smaller vectors due to their relatively big size, electrostatic properties, solubility, and binding to serum proteins [6–8].

**Fig. 1:**
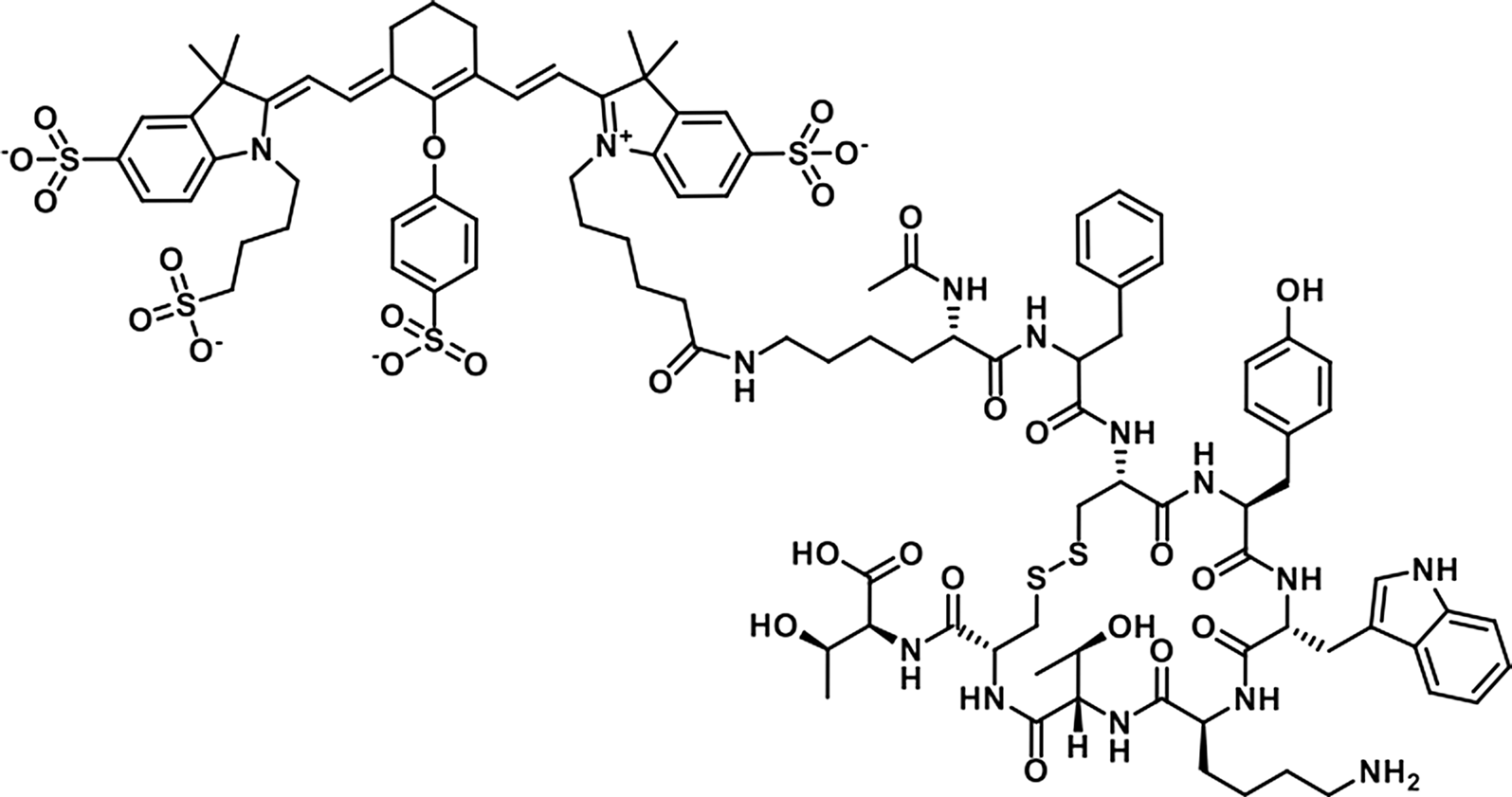
Structure of Ac-Lys^0^(IRDye800CW)-Tyr^3^-octreotate (800CW-TATE)

In our previous work, we found that cyanine dyes such as IRDye800CW specifically bind to cells that lost membrane integrity and thus, to necrotic regions in tumors [9–12]. The presence of necrotic regions is common in fast-growing tumors and necrosis binding of the dye can therefore increase tumor uptake. Here, we describe dead cell-binding of Ac-Lys^0^(IRDye800CW)-Tyr^3^-octreotate (800CW-TATE, Fig. 1), a contrast agent for molecular fluorescence-guided surgery of meningiomas [13]. IRDye800CW is a widely applied fluorescent cyanine dye, which has fluorescent properties in the near-infrared region (NIR, absorption: 774 nm, emission: 789 nm). Tyr^3^-octreotate [14] serves as a targeting vector, able to bind to the somatostatin receptor subtype 2 (SSTR_2_), which is commonly upregulated in neuroendocrine tumors (NET) [15], such as meningioma’s [16]. Tyr^3^-octreotate conjugated to DOTA and labeled with gallium-68 or lutetium-177 is routinely used for diagnosis and radionuclide therapy of NET patients, respectively [17–19].

This study aims to investigate the effect of the IRDye800CW-mediated dead cell-binding on the physiological properties of 800CW-TATE. These dead cell-binding properties can be interfering for routine experiments. For instance, some of the experimental procedures used to study radioactive labeled compounds make use of cells with disrupted cell membranes. These experimental procedures cannot be applied to study the binding of cyanine-labeled agents as the binding to these disrupted cells interferes with the experiment.

## Materials and methods

### In vitro dead cell binding

Human osteosarcoma cells (U2OS), either transfected with SSTR_2_ [20] or wild-type (without SSTR_2_ expression) were cultured in DMEM (Gibco Life Technologies, Waltham, MA, USA) with 10% fetal calf serum and 1% penicillin. The cells were seeded in 24 well plates (10^5^ cells per well) and grown until confluent. The cells were kept alive or killed with 50 µL EtOH (70%) and incubated with 800CW-TATE (10 nM, piCHEM, Grambach, Austria) in culturing medium for 30 min. A blocking experiment was included by co-incubation of 800CW-TATE (10 nM) with DOTA-TATE (10 µM, Bachem, Bubendorf, Switzerland). Control experiments were included by incubation of the cells with a NIR-fluorescent dye that does not bear a cyanine motive: Rhodamine 800 (10 nM, Sigma-Aldrich). Additionally, wells without seeded cells were exposed to PBS or EtOH and then incubated with 800CW-TATE, to investigate non-specific binding to plastic. After gentle washing with PBS, NIR-fluorescence imaging of the whole plate was performed on an Odyssey flatbed scanner (Li-Cor, Lincoln, USA). 800CW-TATE binding was based on fluorescent signal ± SD and each experiment was performed in *n* = 12.

### In vitro dead cell binding [^111^In]In-DOTA-TATE

U2OS cells, either transfected with SSTR_2_ [20] or wild-type were seeded in 24 well plates (10^5^ cells per well) and grown until confluent. The cells were kept alive or killed with 50 µL EtOH (70%) and incubated with [^111^In]In-DOTA-TATE (10 nM, 50 MBq/nmol) in culturing medium for 30 min. A blocking experiment was included by co-incubation with DOTA-TATE (10 µM). After gentle washing with PBS, autoradiography of the whole plate was performed using a super-resolution phosphor screen and a Cyclone® Plus system (Perkin Elmer, Waltham, MA, USA). Then, the cells were detached with NaOH (1 M, 1 mL) and collected in tubes for *γ*-counting (1480 *γ*-counter Perkin Elmer, Waltham, MA, USA). Each experiment was performed in *n* = 6.

### Microscopy

U2OS cells, either transfected with SSTR_2_ [20] or wild-type were seeded on 10 mm coverslips in 24 well plates (10^5^ cells per well). The cells were kept alive or killed with 50 µL EtOH (70%) and incubated in culturing medium with or without 800CW-TATE (100 nM) for 30 min. After PBS washing, the cells were incubated at 37˚C with Calcein AM (1 µM) and Propidium iodide (1 µM) in PBS for 15 min to stain live and dead cells, respectively. The cells were washed with PBS, fixated with PFA (4% in PBS, 20 min) and nuclei were stained with DAPI (Sigma-Aldrich, 1:1000 in PBS, 5 min). After final washing with PBS, the coverslips were removed from the well plate and mounted on a microscope slide for fluorescence microscope imaging using a magnification of 20x.

### Ex vivo experiments

Frozen NCI-H69 and CH-157MN xenografts were sectioned in 10 µm thick slices, which were mounted on Starfrost glass slides (Thermo Fisher). To prevent non-specific binding, fresh sections were incubated for 10 min at room temperature (RT) with washing buffer (167 mM Tris–HCl, 5 mM MgCl_2_) with bovine serum albumin (BSA, 0.25 g/L). Slides were drained and incubated for 1 h at RT with a fixed volume of 100 µL washing buffer, containing 800CW-TATE (100 nM or 1 µM) and BSA (1 g/L). To determine specificity, additional sections were co-incubated with increasing concentrations of DOTA-TATE block (0×, 1×, 5×, 10×, 50×, 100×, 250×, 500×, or 1000× the concentration of 800CW-TATE). The sections were drained, washed, and after drying, placed in an Odyssey CLx (Li-cor Biosciences) for NIR-fluorescence detection. Subsequently, H&E staining was performed on the sections, based on which regions of interest (ROIs) were drawn on the viable sections of the tumors for quantification of the NIR-fluorescence signal using ImageJ.

### In vivo experiments

Tumor tissue from mice described in another publication was used [13]. Briefly, BALB/c-nu mice were inoculated with 5 * 10^6^ human small cell lung cancer cells (NCI-H69) in 1:1 medium: matrigel [13, 14, 21]. When the tumors reached a size of 400 mm^3^, 800CW-TATE (3 µg, 1.36 nmol, 50 µL saline) was injected retro-orbitally. A blocking study was included, for which a mouse received two injections of DOTA-TATE (3 mg, 2.2 µmol, 2 × 50 µL saline) followed by one injection with 800CW-TATE (3 µg, 1.36 nmol, 50 µL saline), with 5 min intervals. Four-hour post-injection, the mice were sacrificed, after which tumors were collected and embedded in paraffin for histological analysis. Adjacent paraffin sections of 4 µm were prepared from the tumors. The sections were imaged for NIR-fluorescence on an Odyssey. Immunofluorescence (IF) staining was performed for SSTR_2_ (SSTR_2_ primary antibody, Abcam; 1:100; secondary antibody goat anti-rabbit Alexa 596, Abcam, 1:1000). Histochemical TUNEL dead cell staining was performed using the In Situ Cell Death Detection Kit, Fluorescein (Roche Diagnostics GmbH, Mannheim, Germany). Stitched fluorescence microscope images of the stained sections were acquired on a fluorescence microscope (TriPath-Imaging, Burlington, USA) equipped with an Aziocam MRm (Zeiss, Oberkochen, Germany) and an Axiocam 208 color (Zeiss) using a magnification of 40×. The Institutional Review Board of the University of Groningen approved the study and animal care complied with the Guide for the Care and Use of Laboratory Animals.

## Results

**Fig. 2:**
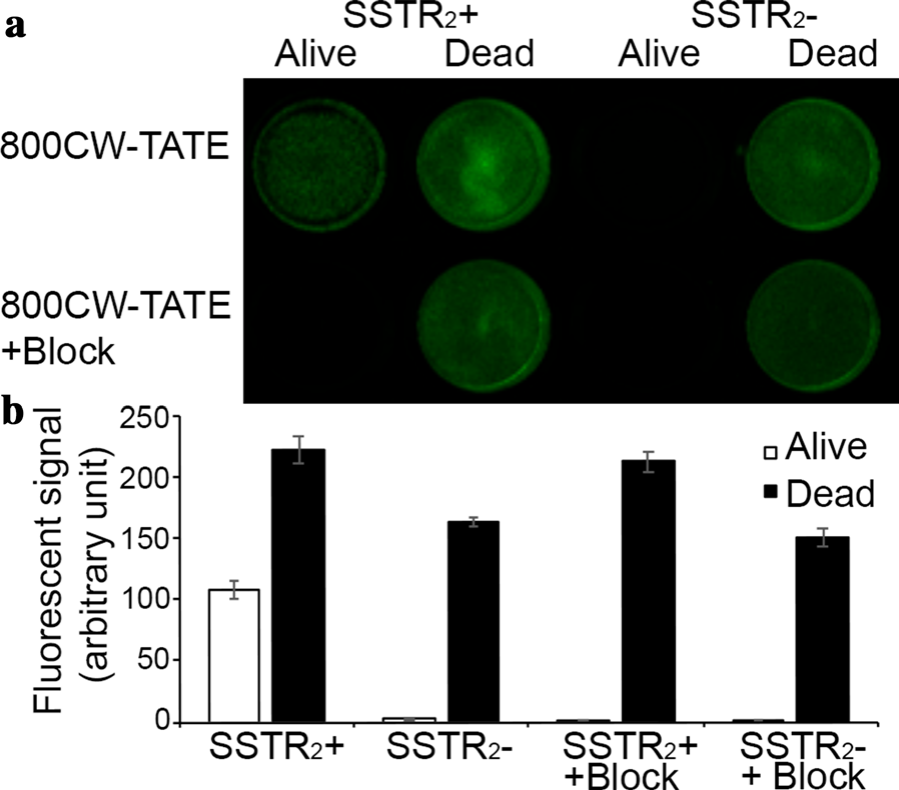
NIR-fluorescence imaging of alive or dead U2OS cells with and without SSTR_2_-expression, exposed to 800CW-TATE (10 nM in culturing medium). Blocking is performed by co-incubation with DOTA-TATE (10 µM). Fluorescent signal quantified ± SD, *n* = 12. SSTR_2_ +  = U2OS cells transfected with SSTR_2_; SSTR_2_- = wild-type U2OS cells without SSTR_2_-expression; 800CW-TATE = Ac-Lys^0^(IRDye800CW)-Tyr^3^-octreotate; DOTA-TATE = DOTA^0^-Tyr^3^-octreotate

In our previous publication, we determined that the IC_50_ values for SSTR_2_ were 72 nM for 800CW-TATE and 2.5 nM for DOTA-TATE [13]. In vitro binding of 800CW-TATE on plated cells was demonstrated (Fig. 2). The four wells on the left contain U2OS cells with stable expression of SSTR_2_ whereas the four wells on the right contain the wild-type U2OS cells which do not express SSTR_2_. The cells were either killed or kept alive before incubation with either 800CW-TATE alone or 800CW-TATE in the presence of an excess of DOTA-TATE. There was SSTR_2_-mediated binding of 800CW-TATE, as it was effectively blocked by DOTA-TATE. What was striking was the strength of the signal observed from the dead cells. It was not mediated by SSTR_2_ since the signal was not blocked by the excess DOTA-TATE. Similarly, fluorescence was observed from the dead cells without SSTR_2_-expression. The dead cell binding was significant as it was twice as high as SSTR_2_-mediated binding (206%, *P* < 0.001). In contrast, [^111^In]In-DOTA-TATE did not showcase dead cell-mediated binding (Additional file 1: Supplementary data 1.1). No 800CW-TATE binding was observed to ethanol exposed wells without cells (Additional file 1: Supplementary data 1.2), therefore excluding binding to the ethanol-exposed plastic of the wells. Moreover, dead and alive cells, with and without SSTR_2_-expression, were exposed to Rhodamine 800, a NIR-fluorescent dye that does not bear a cyanine motive. There was no binding observed of Rhodamine 800, indicating that this dead cell-binding is mediated via the cyanine motive of IRDye800CW (Additional file 1: Supplementary data 1.3). Further confirmation of 800CW-TATE binding to dead cells was obtained from microscopy images. Cells incubated in culturing medium without 800CW-TATE showcased no fluorescence, therefore indicating that the NIR-fluorescent signal is not caused by autofluorescence from dead cells (Additional file 1: Supplementary data 2).

**Fig. 3:**
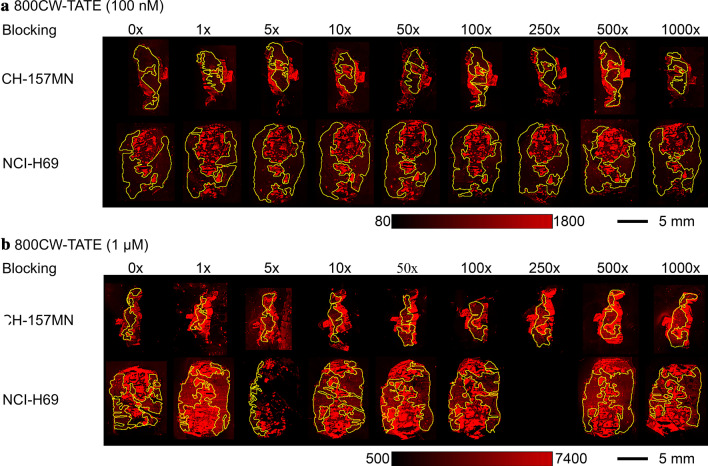
Ex vivo NIR-fluorescence uptake in SSTR_2_-positive NCI-H69 and SSTR_2_-negative CH-157MN cryosections after incubation of 800CW-TATE (**a**: 100 nM; **b**: 1 µM) in the presence of increasing amounts of blocking by DOTA-TATE to block SSTR_2_-mediated binding. In yellow, ROIs of viable tumor regions. The intensity bar indicates fluorescent signal intensity (arbitrary unit)

The NIR-fluorescence uptake in the frozen tumor sections exposed to 800CW-TATE was heterogeneous. Based on H&E staining, viable regions of the tumors were selected to draw ROIs (Additional file 1: Supplementary data 3.1). It was observed that the fluorescent signal was highest in the disrupted regions of the tissue sections. NCI-H69 tumors have a well-established expression of SSTR_2_ whereas CH-157MN are SSTR_2_-negative [13]. However, co-incubation with DOTA-TATE did not block the fluorescence signal from 800CW-TATE in the NCI-H69 sections. Moreover, the CH-157MN sections showcased similar uptake intensities, thereby further confirming that the fluorescence binding to the sections is not SSTR_2_-mediated (Fig. 3). Consequently, the results from ex vivo experiments on NCI-H69 and CH-157MN cryosections were not satisfactory, since the findings were not in line with the results from the in vitro experiment in Fig. 2, where SSTR_2_-mediated binding is effectively blocked from live, SSTR_2_-expressing cells with DOTA-TATE. Quantification of the mean fluorescent uptake is shown in Additional file 1: Supplementary data 3.

**Fig. 4:**
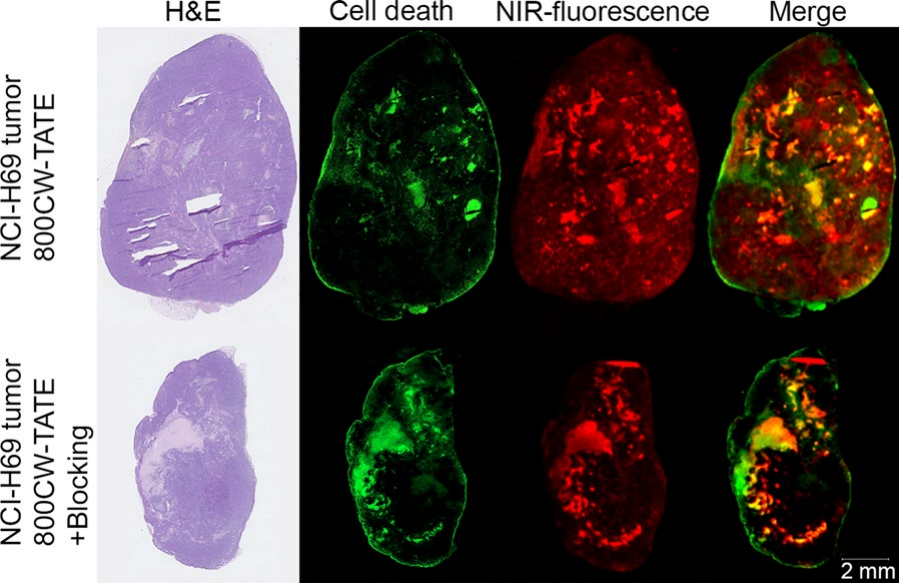
Consecutive sections of paraffin-embedded NCI-H69 tumor xenografts from mice, tumors resected 4 h post-injection with either 3 µg 800CW-TATE (top) or 3 mg DOTA-TATE (as a blocking agent from SSTR_2_) and 3 µg 800CW-TATE (bottom). From left to right are depicted H&E staining, fluorescent cell death staining (TUNEL), NIR-fluorescent signal from 800CW-TATE uptake, and a merging of dead cell staining and NIR-fluorescence

800CW-TATE was studied as a novel contrast agent for molecular, fluorescence-guided, surgery (MFGS) on NCI-H69 tumor xenograft-bearing mice. In this model, the tracer was able to detect tumor tissue for MFGS [13]. To verify SSTR_2_ specific binding to the tumor, a blocking experiment was performed by pre-administration of DOTA-TATE, thereby outcompeting 800CW-TATE for binding to SSTR_2_. It was observed, however, that fluorescence was only partially reduced by DOTA-TATE blocking (reduction of 57.8 ± 5.2%). Moreover, after dissection of the tumors, a heterogeneous uptake of the fluorescent compound was observed in paraffin-embedded sections (Fig. 4, red). Adjacent sections were subjected to H&E staining (Fig. 4), fluorescently stained for cell death using TUNEL staining (Fig. 4, green), or for SSTR_2_-expression (Additional file 1: Supplementary data 4). The top and bottom sections in Fig. 4 represent a tumor from a mouse injected with 800CW-TATE and from the blocking experiment, respectively. A strong overlap of the NIR-fluorescence uptake and the necrotic regions was observed in these tumors. Similar to what we observed with cultured cells in Fig. 2, DOTA-TATE blocked the signal from viable cells, as the NIR-fluorescence signal in the viable regions of the tumors was reduced by DOTA-TATE blocking.

## Discussion

In the current paper, we have shown that the necrosis avidity of cyanine-dyes, such as IRDye800CW, can significantly contribute to the binding behavior of targeting vectors conjugated to cyanines, intended for MFGS. This has some disadvantages and advantages. One disadvantage is that these probes cannot be used in routine protocols frequently used for radionuclide probes to determine specific binding of the probe directly on tissue sections. We showed in this study that these experiments resulted in high amounts of unspecific binding, which can be explained by the fact that in the process of cutting the tissue sections, the cellular membranes in the tissue are compromised. We showed in an earlier publication that cyanine dyes, such as IRDye800CW, bind to endoplasmic proteins that become available when cells lose membrane integrity [9].

When 800CW-TATE is injected systemically in SSTR_2_-expressing tumor-bearing mice, it will bind to SSTR_2_ via the targeting octreotate peptide, but also to necrotic tissue via IRDye800CW. This was also observed in our earlier study using 800CW-2DG and 800CW-EGF, where the targeting molecules bind to their specific target and IRDye800CW to necrosis [9]. 800CW-EGF has a molecular weight of around 7 kD and could still bind to necrosis. IRDye800CW conjugated to a 25 kD polyethyleneglycol (PEG)-chain, on the other hand, could not bind to necrosis, most probably due to steric hindering. This suggests that small molecules or peptides conjugated to IRDye800CW with a molecular weight < 7 kD still retain necrosis avidity [9]. This also explains in vitro the presence of a specific 800CW-TATE signal in live SSTR_2_ expressing U2OS cells and a non-specific signal in dead cells (Fig. 2). The latter was confirmed by the fact that the signal could not be blocked by excess DOTA-TATE and was present in both SSTR_2_-expressing and non-expressing dead cells. Moreover, in the tissue sections of SSTR_2_-positive NCI-H69 tumor-bearing mice, treated with 800CW-TATE (Fig. 4) the NIR-fluorescence signal (red) co-localized (yellow-orange), to a considerable extent with the TUNEL signal (green) indicating dead cells. When an excess of DOTA-TATE was co-administered, the specific signal disappeared but the necrosis specific signal remained.

These findings could also explain retrospectively our previous unexpected results in vivo using a dual labeled octreotate peptide. This peptide was attached to both DTPA as a chelator for indium-111 labeling and the fluorescent cyanine dye Cy5 [21]. We observed that the in vitro affinity of the dual-labeled octreotate dropped by more than a logarithmic unit compared with the peptide only labeled with indium-111. Unexpectedly though, there was no significant difference in overall tumor-uptake in vivo between the dual labeled peptide and the radioactive peptide. We hypothesized that this was due to the blood protein binding of the dye, which is a known property of cyanines [8]. However, the necrosis binding must have influenced the tumor uptake and therefore compensated for the reduced affinity for the target protein.

Most solid tumors develop necrotic tissue, especially in the center of the tumor appearing as a necrotic core [22, 23]. This is due to improper functioning of immature tumor blood vessels and rapid tumor growth leading to insufficient oxygen supply in the center followed by tissue necrosis [24]. This is furthermore correlated with the poor prognosis of the disease [25, 26]. Moreover, we have shown that imaging of necrosis can be used for the early determination of the therapeutic efficacy of irradiation therapy [10] or chemotherapy [11]. For this reason, we recently developed and validated [^111^In]In-DOTA-PEG_4_-800CW, as a specific probe to image tumor necrosis [12].

What could be the advantage for MFGS of using a small molecule or peptide probe that is conjugated with a cyanine dye like IRDye800CW that also targets necrosis? As mentioned before, most solid tumors will develop a necrotic core or necrotic regions at a certain time point depending on the growth rate [24]. The ultimate goal of MFGS is to identify tumor tissue and distinguish it from normal healthy tissue during surgery. Apart from binding to a specific tumor target, binding to necrosis will not interfere with the specific binding. It can actually contribute to the total tumor to background ratio and the intensity of the signal as necrosis is found in most tumors [22]. This will mostly aid tumor detection with dual-labeled probes for pre-operative imaging and initial localization of the tumor during MFGS. Necrosis can lead to a reduced signal intensity from tumor markers for which the dead cell-binding can compensate. For successful fluorescence-guided tumor resection, it is generally important that there is prompt discrimination between the tumor and healthy surrounding tissue [27], for which the dead cell-binding in the tumor center is perhaps less relevant when dealing with solid tumors. In some cases, however, tumors may cause necrosis in the surrounding tissues [28]. In such situations, this dead cell-binding can be highly helpful in removing more diseased tissue that can increase the chance of successful surgery. Regardless, of this benefit, necrotic tissue should be removed as much as possible as it cannot be salvaged, causes inflammatory responses, and even releases tumorigenic factors [22, 29].

## Conclusion

In conclusion, one has to be aware that tumor-targeting molecules or peptides conjugated to a cyanine dye, like IRDye800CW, will also bind to necrosis, that is, intracellular proteins of necrotic cells that become available when cells have lost membrane integrity. This will prevent its use in determining the specific binding of the tumor-targeted probe directly on tissue sections because intracellular proteins will be ubiquitously exposed. When using these tumor-targeted probes in vivo to determine the presence of the specific target, e.g. using NIR-fluorescence imaging or afterward in histological sections, one needs to be aware of possible binding to necrotic tissue. Despite these disadvantages, a great advantage for MFGS could be that binding to its specific target and to necrosis will increase the tumor uptake, contributing to the efficiency of MFGS.

## Supplementary information

**Additional file 1**. Supplementary data 1: controls in vitro dead/alive cell binding. Supplementary data 2: microscopy. Supplementary data 3: ex vivo binding of 800CW-TATE and to NCI-H69 and CH-157MN tumor sections. Supplementary data 4: SSTR_2_ IF staining.

## Data Availability

The data that support the findings of this study are available from the corresponding author on reasonable request.

## Abbreviations

800CW-TATE: Ac-Lys^0^(IRDye800CW)-Tyr^3^-octreotate
BSA: Bovine serum albumin
DOTA-TATE: DOTA^0^-Tyr^3^-octreotate
IF: Immunofluorescence
MFGS: Molecular fluorescence-guided surgery
NET: Neuroendocrine tumors
NIR: Near-infrared
PBS: Phosphate buffered saline
PEG: Polyethyleneglycol
SSTR_2_: Somatostatin receptor subtype 2
