# Supplementary material for: Necrosis binding of Ac-Lys0(IRDye800CW)-Tyr3-octreotate: a consequence from cyanine-labeling of small molecules
Source: EJNMMI Res. 2021 May 10;11:47. doi: 10.1186/s13550-021-00789-4 (PMC8110618; doi:10.1186/s13550-021-00789-4)
Supplement: Supplementary file 1 — Additional file 1. Supplementary data 1: controls in vitro dead/alive cell binding. Supplementary data 2: microscopy. Supplementary data 3: ex vivo binding of 800CW-TATE and to NCI-H69 and CH-157MN tumor sections. Supplementary data 4: SSTR2 IF staining. [file 13550_2021_789_MOESM1_ESM.pdf]

Necrosis Binding of Ac-Lys^0(IRDye800CW)-Tyr^3-octreotate: Pros and Cons of Using Cyanine-labeled Small Molecules.

Marcus C.M. Stroet[1,2], Bianca M. Dijkstra[3], Sebastiaan E. Dulfer[3], Schelto Kruijff[4], Wilfred F.A. den Dunnen[5], Frank A.E. Kruyt[6], Rob J.M. Groen[3], Yann Seimbille[1], Kranthi M. Panth[1,2], Laura Mezzanotte[1,2], Clemens W.G.M. Lowik[1,7,#], Marion de Jong[1,#]

1. Department of Radiology and Nuclear Medicine, Erasmus MC, Rotterdam
2. Department of Molecular Genetics, Erasmus MC, Rotterdam.
3. Department of Neurosurgery, University of Groningen, University Medical Center Groningen, the Netherlands
4. Department of Surgery, University of Groningen, University Medical Center Groningen, Groningen, the Netherlands
5. Department of Pathology and Medical Biology, University of Groningen, University Medical Center Groningen, Groningen, the Netherlands
6. Department of Medical Oncology, University of Groningen, University Medical Center Groningen, the Netherlands
7. CHUV Department of Oncology, University of Lausanne, Switzerland

#. Both authors are senior author and share last authorship.

Supplementary data 1: controls *in vitro* dead/alive cell binding

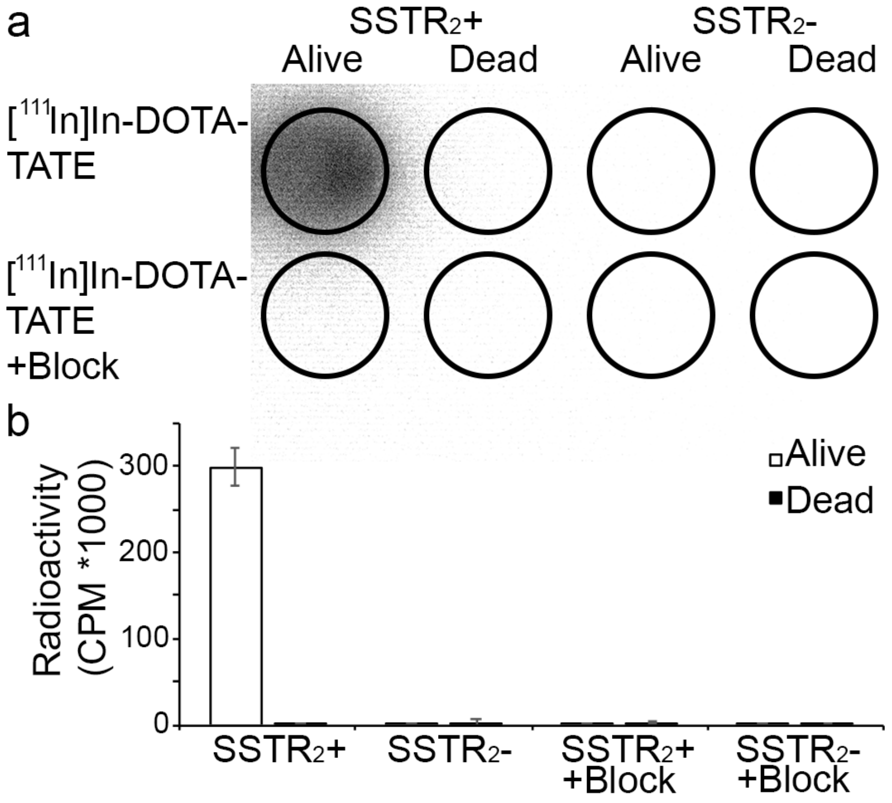

Supplementary figure 1.1: Radioactivity visualization and quantification of alive or dead U2OS cells with and without $SSTR_2$-expression, exposed to [^{111}In]In-DOTA-TATE (50 MBq/nmol, 10 nM in culturing medium). Blocking is performed by co-incubation with DOTA-TATE (10 µM). a: autoradiography of well plate after washing and b: Amount of radioactivity collected from the wells and quantified by γ-counting. Radioactive signal quantified in counts per minute ± SD, n=6. $SSTR_2$+ = U2OS cells transfected with $SSTR_2$; $SSTR_2$- = wild-type U2OS cells without $SSTR_2$-expression; DOTA-TATE = DOTA^0-Tyr^3-octreotate

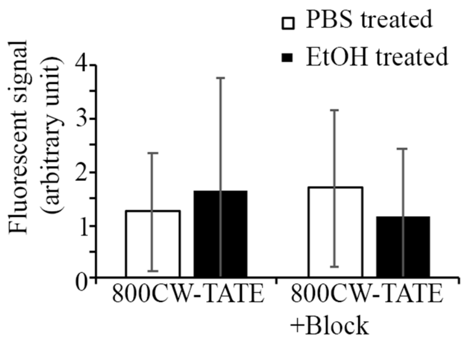

Supplementary figure 1.2: Quantification of NIR fluorescent signal from empty wells, exposed to 800CW-TATE after treatment with PBS or EtOH.

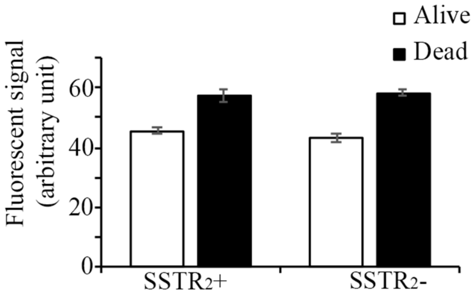

Supplementary figure 1.3: Quantification of NIR fluorescent signal from alive or dead U2OS cells with and without $SSTR_2$-expression, exposed to Rhodamine-800. Fluorescence recorded on the 700 nm channel of an Odyssey. Fluorescent signal quantified ± SD, n=12. $SSTR_2$+ = U2OS cells transfected with $SSTR_2$; $SSTR_2$- = wild-type U2OS cells without $SSTR_2$-expression

Supplementary data 2: microscopy

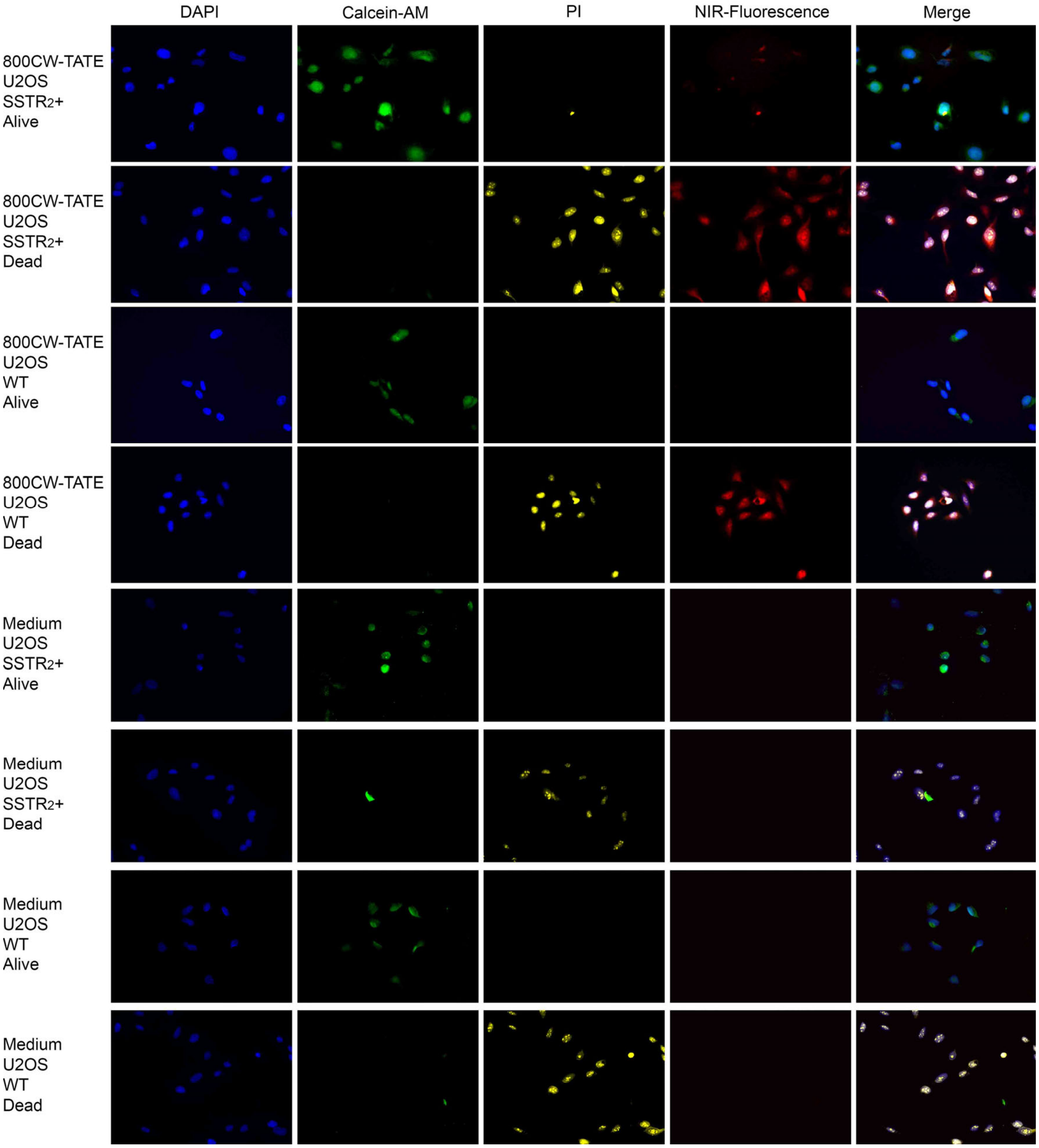

Supplementary figure 2: Fluorescence microscopy images of dead and alive cells incubated in culturing medium with and without 800CW-TATE (30 min 100 nM). 800CW-TATE binding to dead cells and $SSTR_2$ expressing cells (red). Nuclei stained with DAPI (blue), alive cells stained with Calcein-AM (green), dead cells stained with propidium-iodide (PI, yellow).

Supplementary data 3: *ex vivo* binding of 800CW-TATE and to NCI-H69 and CH-157MN tumor sections

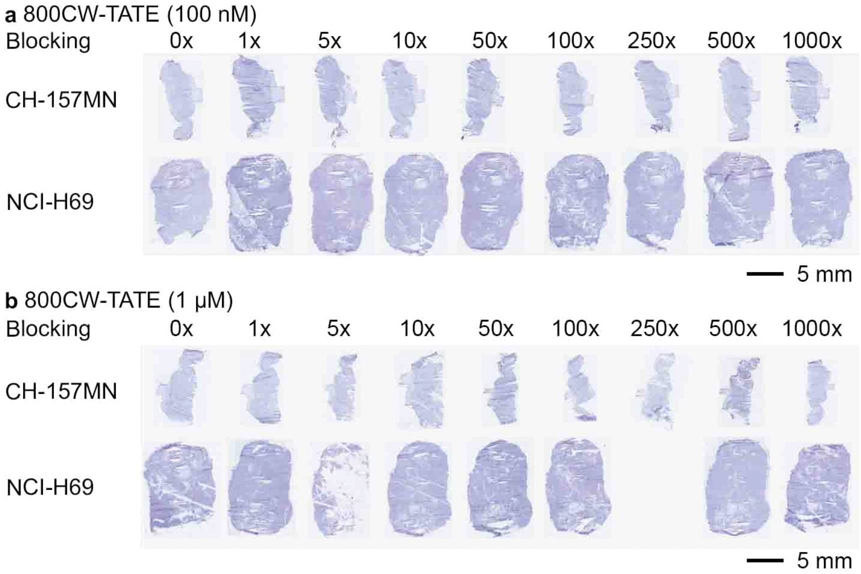

Supplementary figure 3.1: $SSTR_2$-positive NCI-H69 and $SSTR_2$-negative CH-157MN cryosections displayed in figure 3 after H&E staining.

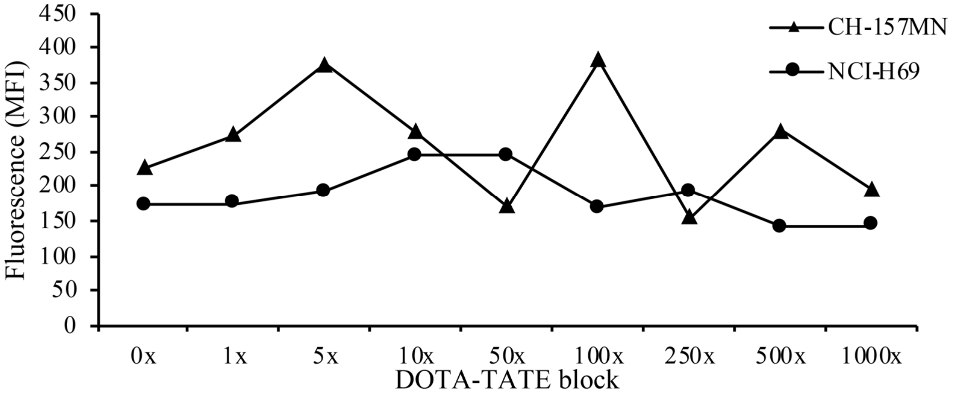

Supplementary figure 3.2: Quantification of mean fluorescent intensity (MFI) from viable regions of frozen tumor sections, incubated with 800CW-TATE (100 nM) in the presence of increasing concentrations of DOTA-TATE to block $SSTR_2$-mediated binding. 800CW-TATE = Ac-Lys^0(IRDye800CW)-Tyr^3-octreotate; DOTA-TATE = $DOTA^0$-Tyr^3-octreotate; Dots represent CH-157MN ($SSTR_2$ negative tumor); triangles represent NCI-H69 ($SSTR_2$ positive tumor). Fluorescent images depicted in figure 3a.

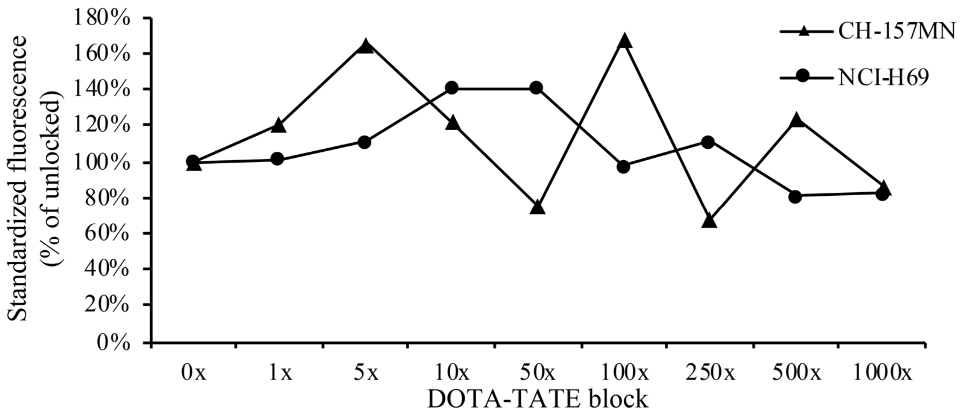

Supplementary figure 3.3: Standardized fluorescence uptake as a percentage of unblocked from viable regions of frozen tumor sections, incubated with 800CW-TATE (100 nM) in the presence of increasing concentrations of DOTA-TATE to block $SSTR_2$-mediated binding. Dots represent CH-157MN ($SSTR_2$ negative tumor); triangles represent NCI-H69 ($SSTR_2$ positive tumor).

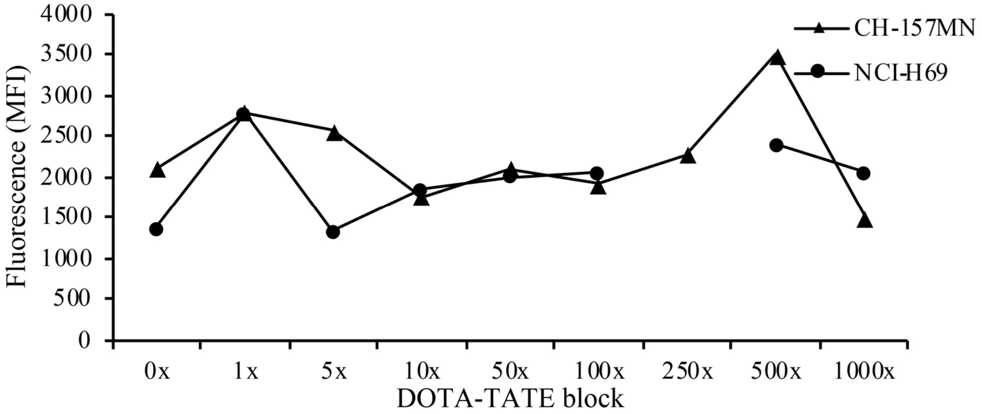

Supplementary figure 3.4: Quantification of MFI from viable regions of frozen tumor sections, incubated with 800CW-TATE (1 μM) in the presence of increasing concentrations of DOTA-TATE to block $SSTR_2$-mediated binding. 800CW-TATE = Ac-Lys^0(IRDye800CW)-Tyr^3-octreotate; DOTA-TATE = $DOTA^0$-Tyr^3-octreotate; Dots represent CH-157MN ($SSTR_2$ negative tumor); triangles represent NCI-H69 ($SSTR_2$ positive tumor). Fluorescent images depicted in figure 3b.

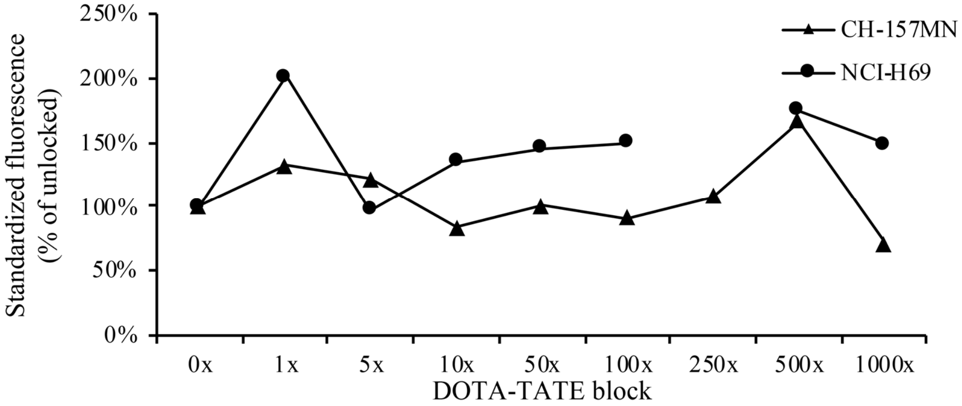

Supplementary figure 3.5: Standardized fluorescence uptake as a percentage of unblocked from viable regions of frozen tumor sections, incubated with 800CW-TATE (1 μM) in the presence of increasing concentrations of DOTA-TATE to block $SSTR_2$-mediated binding. Dots represent CH-157MN ($SSTR_2$ negative tumor); triangles represent NCI-H69 ($SSTR_2$ positive tumor).

Supplementary data 4: $SSTR_2$ IF staining

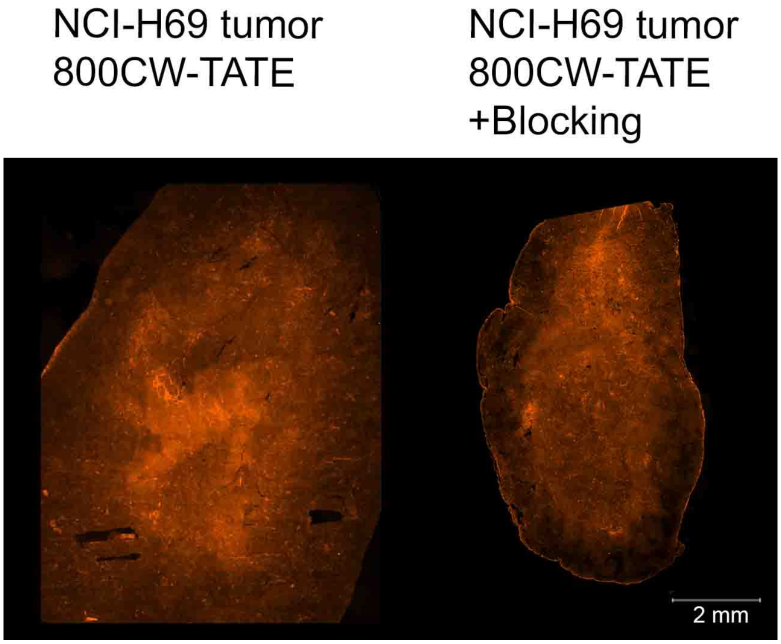

Supplementary figure 4: Tiled microscopy images of consecutive sections from paraffin-embedded NCI-H69 tumor xenografts from figure 4, after IF staining for $SSTR_2$-expression.
